# 2,2,2-Trifluoro-1-[3-(2,2,2-trifluoro­acet­yl)azulen-1-yl]ethanone

**DOI:** 10.1107/S1600536811017569

**Published:** 2011-05-14

**Authors:** Sebastian Förster, Frank Eissmann, Wilhelm Seichter, Edwin Weber

**Affiliations:** a Institut für Organische Chemie, TU Bergakademie Freiberg, Leipziger Strasse 29, D-09596 Freiberg/Sachsen, Germany

## Abstract

There are two mol­ecules in the asymmetric unit of the title compound, C_14_H_6_F_6_O_2_, in which the azulene systems possess an almost planar geometry with maximum deviations of 0.0438 (15) and 0.0396 (14) Å. Besides intra- and inter­molecular C—H⋯O and C—H⋯F inter­actions, the structure displays three F⋯F contacts [2.793 (2), 2.8820 (17) and 2.9181 (16) Å]. Furthermore, a characteristic azulene π-stacking is observed with an alternating sequence of electron-rich five-membered rings and electron-deficient seven-membered rings [centroid–centroid distances = 3.5413 (12), 3.6847 (12), 3.5790 (12) and 3.7718 (12) Å].

## Related literature

For the synthesis, see: Mathias & Overberger (1980 ▶); Zielinski *et al.* (2008 ▶). For the crystal structure of the parent azulene, see: Robertson *et al.* (1962 ▶). For halogen inter­actions in mol­ecular crystal structures, see: Brammer *et al.* (2001 ▶); Metrangolo *et al.* (2008 ▶). 
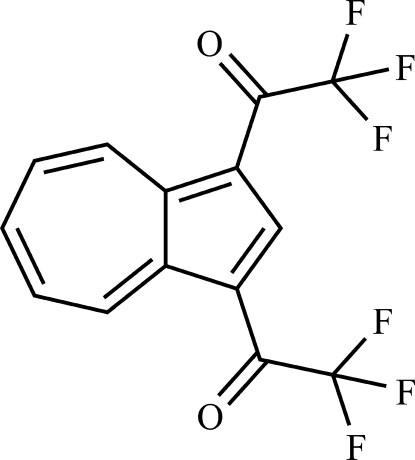


## Experimental

### 

#### Crystal data

C_14_H_6_F_6_O_2_
*M* *_r_* = 320.19
Triclinic, 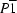

*a* = 7.1634 (2) Å
*b* = 10.8681 (4) Å
*c* = 16.3286 (5) Å
α = 81.544 (2)°
β = 83.310 (2)°
γ = 80.009 (2)°
*V* = 1232.92 (7) Å^3^
*Z* = 4
Mo *K*α radiation
μ = 0.18 mm^−1^
*T* = 100 K
0.40 × 0.14 × 0.07 mm

#### Data collection

Bruker APEXII CCD area-detector diffractometer
Absorption correction: multi-scan (*SADABS*; Bruker, 2007 ▶) *T*_min_ = 0.933, *T*_max_ = 0.988
20089 measured reflections
5321 independent reflections
3680 reflections with *I* > 2σ(*I*)
*R*_int_ = 0.031

#### Refinement

*R*[*F* ^2^ > 2σ(*F* ^2^)] = 0.038
*wR*(*F* ^2^) = 0.106
*S* = 1.02
5321 reflections
397 parameters
H-atom parameters constrained
Δρ_max_ = 0.25 e Å^−3^
Δρ_min_ = −0.31 e Å^−3^

### 

Data collection: *APEX2* (Bruker, 2007 ▶); cell refinement: *SAINT* (Bruker, 2007 ▶); data reduction: *SAINT*; program(s) used to solve structure: *SHELXS97* (Sheldrick, 2008 ▶); program(s) used to refine structure: *SHELXL97* (Sheldrick, 2008 ▶); molecular graphics: *SHELXTL* (Sheldrick, 2008 ▶); software used to prepare material for publication: *SHELXTL* and *PLATON* (Spek, 2009) ▶.

## Supplementary Material

Crystal structure: contains datablocks I, global. DOI: 10.1107/S1600536811017569/im2284sup1.cif

Structure factors: contains datablocks I. DOI: 10.1107/S1600536811017569/im2284Isup2.hkl

Supplementary material file. DOI: 10.1107/S1600536811017569/im2284Isup3.mol

Supplementary material file. DOI: 10.1107/S1600536811017569/im2284Isup4.cml

Additional supplementary materials:  crystallographic information; 3D view; checkCIF report

## Figures and Tables

**Table 1:** Hydrogen-bond geometry (Å, °)

*D*—H⋯*A*	*D*—H	H⋯*A*	*D*⋯*A*	*D*—H⋯*A*
C2—H2⋯F3	0.95	2.40	2.916 (2)	114
C2*A*—H2*A*⋯F3*A*	0.95	2.45	2.972 (2)	115
C2*A*—H2*A*⋯F5*A*	0.95	2.50	2.968 (2)	111
C5—H5⋯O2	0.95	2.31	2.988 (2)	127
C5*A*—H5*A*⋯O2*A*	0.95	2.33	3.001 (2)	127
C6—H6⋯O2*A*^i^	0.95	2.52	3.236 (2)	133
C6*A*—H6*A*⋯O2^i^	0.95	2.47	3.160 (2)	130
C8—H8⋯F2*A*^ii^	0.95	2.45	3.358 (2)	160
C9—H9⋯O1	0.95	2.31	2.983 (2)	127
C9—H9⋯O1*A*^ii^	0.95	2.58	3.454 (2)	153
C9*A*—H9*A*⋯O1^ii^	0.95	2.50	3.352 (2)	149
C9*A*—H9*A*⋯O1*A*	0.95	2.31	2.993 (2)	128

Symmetry codes: (i) 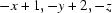; (ii) 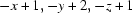.

**Fig. 1.**
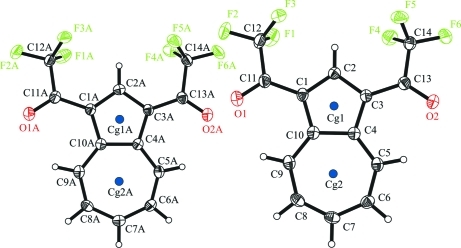
Asymmetric unit of the title compound, showing the atom numbering schemes. Displacement ellipsoids are drawn at the 50% probability level.

**Fig. 2.**
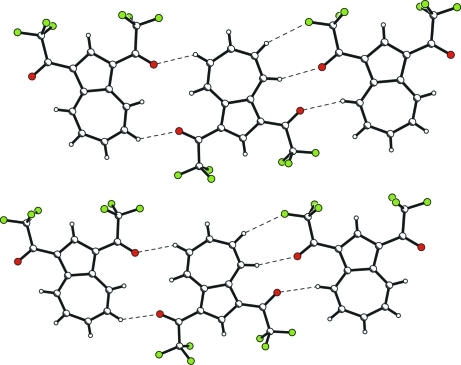
Molecular sheets within the packing of the title compound. Intermolecular interactions are represented as dashed lines.

**Fig. 3.**
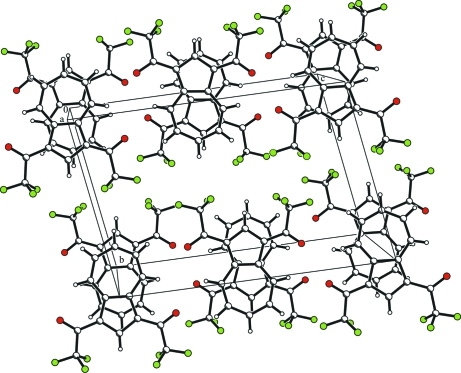
Packing diagram viewed down the a axis, showing the stacking interactions of the azulene systems.

## supplementary crystallographic information

### Comment

The asymmetric unit of the crystal structure contains two molecules featuring planar azulene ring systems (Fig 1). In the crystallographic *b* and *c* directions the structure is stabilized by hydrogen bonding both between the carbonyl oxygen or fluorine atoms and hydrogen atoms of the electronic deficient seven-membered ring (Fig 2). The crystal structure is characterized by formation of molecular stacks along the crystallographic *a*-axis (Fig 3). Due to the inherent dipole character of the azulene ring system, the molecules are arranged in a head-to-tail fashion within the stacks. Each molecule in the asymmetric unit shows two different interactions to its stack neighbors, which leads to four different distances between the centroids *Cg*1···*Cg*2^i^, *d* = 3.5413 (12), *Cg*1···*Cg*2^ii^, *d* = 3.6847 (12), *Cg*1A···*Cg*2A^iii^, *d* = 3.5790 (12), *Cg*1A···*Cg*2A^iv^, *d* = 3.7718 (12). Furthermore, three halogen···halogen contacts were observed: F5···F5 [*d* = 2.7931 (23) Å, *θ*_1_ = *θ*_2_ = 145.79 (14)°], F3···F6A [*d* = 2.8820 (17), *θ*_1_ = 124.02 (11), *θ*_2_ = 113.52 (12)] and F6A···F6 [*d* = 2.9181 (16), *θ*_1_ = 161.89 (11), *θ*_2_ = 156.90 (12)]. Symmetry codes: (i) = -*x*, 2 - *y*, -*z*; (ii) = 1 - *x*, 2 - *y*, -*z*; (iii) 1 - *x*, 2 - *y*, 1 - *z*; (iv) 2 - *x*, 2 - *y*, 1 - *z*.

### Experimental

The title compound was prepared according to the literature procedure of Mathias *et al.* (1980). Crystallization by slow evaporation from acetone yielded suitable crystals after 3 days.

### Refinement

Aromatic H atoms were positioned geometrically and allowed to ride on their respective parent atoms, with C—H = 0.95 Å and *U*_iso_ = 1.2 *U*_eq_(C).

### Crystal data

**Table:**

C_14_H_6_F_6_O_2_	*Z* = 4
*M**_r_* = 320.19	*F*(000) = 640
Triclinic, *P*1	*D*_x_ = 1.725 Mg m^−^^3^
Hall symbol: -P 1	Mo *K*α radiation, λ = 0.71073 Å
*a* = 7.1634 (2) Å	Cell parameters from 6112 reflections
*b* = 10.8681 (4) Å	θ = 2.9–28.9°
*c* = 16.3286 (5) Å	µ = 0.18 mm^−^^1^
α = 81.544 (2)°	*T* = 100 K
β = 83.310 (2)°	Rod, red
γ = 80.009 (2)°	0.40 × 0.14 × 0.07 mm
*V* = 1232.92 (7) Å^3^	

### Data collection

**Table:**

Bruker APEXII CCD area-detector diffractometer	5321 independent reflections
Radiation source: fine-focus sealed tube	3680 reflections with *I* > 2σ(*I*)
graphite	*R*_int_ = 0.031
φ and ω scans	θ_max_ = 27.1°, θ_min_ = 1.3°
Absorption correction: multi-scan (*SADABS*; Bruker, 2007)	*h* = −9→9
*T*_min_ = 0.933, *T*_max_ = 0.988	*k* = −13→13
20089 measured reflections	*l* = −20→20

### Refinement

**Table:**

Refinement on *F*^2^	Primary atom site location: structure-invariant direct methods
Least-squares matrix: full	Secondary atom site location: difference Fourier map
*R*[*F*^2^ > 2σ(*F*^2^)] = 0.038	Hydrogen site location: inferred from neighbouring sites
*wR*(*F*^2^) = 0.106	H-atom parameters constrained
*S* = 1.02	*w* = 1/[σ^2^(*F*_o_^2^) + (0.0465*P*)^2^ + 0.5715*P*] where *P* = (*F*_o_^2^ + 2*F*_c_^2^)/3
5321 reflections	(Δ/σ)_max_ < 0.001
397 parameters	Δρ_max_ = 0.25 e Å^−^^3^
0 restraints	Δρ_min_ = −0.31 e Å^−^^3^

### Special details

**Table:**

Geometry. All e.s.d.'s (except the e.s.d. in the dihedral angle between two l.s. planes) are estimated using the full covariance matrix. The cell e.s.d.'s are taken into account individually in the estimation of e.s.d.'s in distances, angles and torsion angles; correlations between e.s.d.'s in cell parameters are only used when they are defined by crystal symmetry. An approximate (isotropic) treatment of cell e.s.d.'s is used for estimating e.s.d.'s involving l.s. planes.
Refinement. Refinement of *F*^2^ against ALL reflections. The weighted *R*-factor *wR* and goodness of fit *S* are based on *F*^2^, conventional *R*-factors *R* are based on *F*, with *F* set to zero for negative *F*^2^. The threshold expression of *F*^2^ > σ(*F*^2^) is used only for calculating *R*-factors(gt) *etc*. and is not relevant to the choice of reflections for refinement. *R*-factors based on *F*^2^ are statistically about twice as large as those based on *F*, and *R*- factors based on ALL data will be even larger.

### Fractional atomic coordinates and isotropic or equivalent isotropic displacement parameters (Å^2^)

**Table:**

	*x*	*y*	*z*	*U*_iso_*/*U*_eq_	
F1	0.39463 (18)	0.60356 (13)	0.19502 (8)	0.0398 (3)	
F2	0.2168 (2)	0.64770 (12)	0.30613 (7)	0.0412 (3)	
F3	0.09017 (18)	0.61716 (11)	0.19949 (7)	0.0321 (3)	
F4	0.36566 (18)	0.57475 (11)	−0.07337 (8)	0.0346 (3)	
F5	0.05955 (18)	0.59590 (11)	−0.05863 (7)	0.0344 (3)	
F6	0.2086 (2)	0.60122 (12)	−0.18071 (7)	0.0370 (3)	
O1	0.2103 (2)	0.88041 (15)	0.24264 (9)	0.0379 (4)	
O2	0.1713 (2)	0.84185 (14)	−0.17976 (8)	0.0319 (4)	
C1	0.2215 (3)	0.84447 (19)	0.10164 (11)	0.0204 (4)	
C2	0.2071 (3)	0.76448 (18)	0.04375 (11)	0.0195 (4)	
H2	0.1961	0.6778	0.0568	0.023*	
C3	0.2114 (3)	0.83168 (18)	−0.03648 (11)	0.0192 (4)	
C4	0.2344 (2)	0.95792 (18)	−0.03047 (11)	0.0188 (4)	
C5	0.2515 (3)	1.05229 (18)	−0.09710 (12)	0.0213 (4)	
H5	0.2413	1.0298	−0.1503	0.026*	
C6	0.2813 (3)	1.17487 (19)	−0.09636 (12)	0.0237 (4)	
H6	0.2861	1.2254	−0.1490	0.028*	
C7	0.3050 (3)	1.23385 (19)	−0.02918 (13)	0.0246 (4)	
H7	0.3251	1.3189	−0.0424	0.030*	
C8	0.3033 (3)	1.18607 (19)	0.05439 (13)	0.0240 (4)	
H8	0.3249	1.2427	0.0901	0.029*	
C9	0.2749 (3)	1.06782 (19)	0.09354 (12)	0.0223 (4)	
H9	0.2777	1.0549	0.1522	0.027*	
C10	0.2428 (3)	0.96584 (18)	0.05790 (11)	0.0194 (4)	
C11	0.2178 (3)	0.8089 (2)	0.19135 (12)	0.0243 (4)	
C12	0.2291 (3)	0.6679 (2)	0.22329 (12)	0.0280 (5)	
C13	0.1949 (3)	0.78107 (19)	−0.11202 (12)	0.0223 (4)	
C14	0.2074 (3)	0.6369 (2)	−0.10652 (12)	0.0257 (5)	
F1A	0.82283 (17)	0.62221 (12)	0.68301 (8)	0.0367 (3)	
F2A	0.6196 (2)	0.67680 (12)	0.78398 (7)	0.0382 (3)	
F3A	0.52235 (17)	0.63290 (11)	0.67388 (7)	0.0307 (3)	
F4A	0.86640 (18)	0.55033 (11)	0.42077 (8)	0.0373 (3)	
F5A	0.56351 (17)	0.59936 (12)	0.41519 (8)	0.0346 (3)	
F6A	0.7480 (2)	0.57401 (12)	0.30349 (8)	0.0413 (3)	
O1A	0.6058 (2)	0.90591 (14)	0.71153 (8)	0.0314 (4)	
O2A	0.7788 (2)	0.80970 (14)	0.29118 (8)	0.0320 (4)	
C1A	0.6844 (3)	0.85148 (18)	0.57393 (11)	0.0185 (4)	
C2A	0.6948 (3)	0.76475 (18)	0.51849 (11)	0.0193 (4)	
H2A	0.6756	0.6797	0.5335	0.023*	
C3A	0.7379 (3)	0.82104 (18)	0.43672 (11)	0.0187 (4)	
C4A	0.7618 (2)	0.94807 (18)	0.44046 (11)	0.0175 (4)	
C5A	0.8140 (3)	1.03354 (18)	0.37289 (12)	0.0203 (4)	
H5A	0.8301	1.0040	0.3202	0.024*	
C6A	0.8452 (3)	1.15565 (18)	0.37253 (12)	0.0217 (4)	
H6A	0.8807	1.1984	0.3194	0.026*	
C7A	0.8324 (3)	1.22506 (18)	0.43871 (12)	0.0218 (4)	
H7A	0.8577	1.3089	0.4243	0.026*	
C8A	0.7876 (3)	1.18895 (18)	0.52299 (12)	0.0212 (4)	
H8A	0.7898	1.2507	0.5583	0.025*	
C9A	0.7401 (3)	1.07489 (18)	0.56275 (12)	0.0203 (4)	
H9A	0.7133	1.0694	0.6214	0.024*	
C10A	0.7267 (2)	0.96792 (17)	0.52800 (11)	0.0174 (4)	
C11A	0.6426 (3)	0.82740 (19)	0.66380 (12)	0.0220 (4)	
C12A	0.6515 (3)	0.6882 (2)	0.70151 (12)	0.0254 (4)	
C13A	0.7520 (3)	0.76079 (19)	0.36250 (12)	0.0223 (4)	
C14A	0.7319 (3)	0.6197 (2)	0.37514 (13)	0.0271 (5)	

### Atomic displacement parameters (Å^2^)

**Table:**

	*U*^11^	*U*^22^	*U*^33^	*U*^12^	*U*^13^	*U*^23^
F1	0.0358 (7)	0.0393 (8)	0.0377 (7)	0.0061 (6)	−0.0025 (6)	0.0019 (6)
F2	0.0649 (9)	0.0404 (8)	0.0172 (6)	−0.0087 (7)	−0.0069 (6)	0.0030 (5)
F3	0.0401 (7)	0.0298 (7)	0.0280 (7)	−0.0146 (6)	−0.0019 (5)	0.0006 (5)
F4	0.0390 (7)	0.0265 (7)	0.0354 (7)	0.0019 (6)	−0.0017 (6)	−0.0048 (5)
F5	0.0405 (8)	0.0299 (7)	0.0340 (7)	−0.0169 (6)	0.0104 (6)	−0.0059 (5)
F6	0.0578 (9)	0.0325 (7)	0.0242 (7)	−0.0138 (6)	0.0020 (6)	−0.0121 (5)
O1	0.0591 (11)	0.0384 (9)	0.0216 (8)	−0.0204 (8)	−0.0042 (7)	−0.0064 (7)
O2	0.0502 (10)	0.0284 (8)	0.0186 (8)	−0.0108 (7)	−0.0062 (7)	−0.0003 (6)
C1	0.0179 (9)	0.0263 (11)	0.0178 (10)	−0.0050 (8)	−0.0018 (7)	−0.0034 (8)
C2	0.0168 (9)	0.0215 (10)	0.0199 (10)	−0.0039 (8)	−0.0011 (7)	−0.0015 (8)
C3	0.0172 (9)	0.0225 (10)	0.0182 (9)	−0.0039 (8)	−0.0009 (7)	−0.0033 (7)
C4	0.0131 (9)	0.0228 (10)	0.0205 (10)	−0.0030 (7)	−0.0013 (7)	−0.0031 (8)
C5	0.0174 (10)	0.0264 (11)	0.0206 (10)	−0.0036 (8)	−0.0029 (7)	−0.0044 (8)
C6	0.0213 (10)	0.0245 (11)	0.0248 (11)	−0.0054 (8)	−0.0031 (8)	0.0011 (8)
C7	0.0198 (10)	0.0203 (11)	0.0342 (12)	−0.0040 (8)	−0.0026 (8)	−0.0038 (8)
C8	0.0199 (10)	0.0235 (11)	0.0310 (11)	−0.0040 (8)	−0.0048 (8)	−0.0086 (8)
C9	0.0168 (9)	0.0285 (11)	0.0228 (10)	−0.0029 (8)	−0.0052 (8)	−0.0055 (8)
C10	0.0130 (9)	0.0250 (11)	0.0206 (10)	−0.0035 (8)	−0.0015 (7)	−0.0032 (8)
C11	0.0239 (10)	0.0310 (12)	0.0196 (10)	−0.0082 (9)	−0.0030 (8)	−0.0033 (8)
C12	0.0336 (12)	0.0314 (12)	0.0179 (10)	−0.0046 (9)	−0.0029 (8)	0.0000 (8)
C13	0.0216 (10)	0.0261 (11)	0.0195 (10)	−0.0061 (8)	0.0017 (8)	−0.0044 (8)
C14	0.0318 (11)	0.0277 (11)	0.0182 (10)	−0.0078 (9)	0.0034 (8)	−0.0055 (8)
F1A	0.0294 (7)	0.0335 (7)	0.0415 (8)	−0.0003 (6)	−0.0045 (6)	0.0092 (6)
F2A	0.0566 (9)	0.0413 (8)	0.0189 (6)	−0.0200 (7)	−0.0048 (6)	0.0043 (5)
F3A	0.0363 (7)	0.0295 (7)	0.0293 (7)	−0.0159 (5)	−0.0057 (5)	0.0010 (5)
F4A	0.0389 (7)	0.0231 (7)	0.0484 (8)	−0.0008 (6)	−0.0043 (6)	−0.0048 (6)
F5A	0.0330 (7)	0.0371 (8)	0.0383 (7)	−0.0175 (6)	0.0070 (5)	−0.0142 (6)
F6A	0.0581 (9)	0.0388 (8)	0.0341 (7)	−0.0221 (7)	0.0097 (6)	−0.0217 (6)
O1A	0.0429 (9)	0.0332 (9)	0.0212 (7)	−0.0150 (7)	0.0010 (6)	−0.0065 (6)
O2A	0.0481 (10)	0.0293 (8)	0.0194 (8)	−0.0081 (7)	−0.0021 (7)	−0.0043 (6)
C1A	0.0158 (9)	0.0224 (10)	0.0180 (9)	−0.0038 (8)	−0.0034 (7)	−0.0027 (7)
C2A	0.0156 (9)	0.0191 (10)	0.0229 (10)	−0.0030 (7)	−0.0038 (7)	−0.0001 (8)
C3A	0.0167 (9)	0.0219 (10)	0.0184 (9)	−0.0039 (8)	−0.0022 (7)	−0.0043 (8)
C4A	0.0130 (9)	0.0209 (10)	0.0181 (9)	−0.0014 (7)	−0.0034 (7)	−0.0011 (7)
C5A	0.0177 (9)	0.0245 (11)	0.0185 (9)	−0.0017 (8)	−0.0023 (7)	−0.0042 (8)
C6A	0.0190 (10)	0.0247 (11)	0.0204 (10)	−0.0042 (8)	−0.0016 (8)	0.0012 (8)
C7A	0.0176 (10)	0.0207 (10)	0.0270 (11)	−0.0034 (8)	−0.0047 (8)	−0.0009 (8)
C8A	0.0179 (10)	0.0201 (10)	0.0267 (11)	−0.0024 (8)	−0.0043 (8)	−0.0060 (8)
C9A	0.0150 (9)	0.0266 (11)	0.0195 (10)	−0.0018 (8)	−0.0036 (7)	−0.0043 (8)
C10A	0.0118 (9)	0.0220 (10)	0.0186 (9)	−0.0025 (7)	−0.0021 (7)	−0.0026 (7)
C11A	0.0186 (10)	0.0297 (11)	0.0193 (10)	−0.0082 (8)	−0.0026 (7)	−0.0024 (8)
C12A	0.0286 (11)	0.0308 (12)	0.0179 (10)	−0.0098 (9)	−0.0031 (8)	0.0004 (8)
C13A	0.0203 (10)	0.0262 (11)	0.0217 (10)	−0.0051 (8)	−0.0022 (8)	−0.0054 (8)
C14A	0.0298 (11)	0.0284 (12)	0.0256 (11)	−0.0079 (9)	0.0021 (9)	−0.0119 (9)

### Geometric parameters (Å, °)

**Table:**

F1—C12	1.337 (2)	F1A—C12A	1.336 (2)
F2—C12	1.333 (2)	F2A—C12A	1.330 (2)
F3—C12	1.337 (2)	F3A—C12A	1.338 (2)
F4—C14	1.343 (2)	F4A—C14A	1.342 (3)
F5—C14	1.341 (2)	F5A—C14A	1.341 (2)
F6—C14	1.324 (2)	F6A—C14A	1.322 (2)
O1—C11	1.214 (2)	O1A—C11A	1.214 (2)
O2—C13	1.216 (2)	O2A—C13A	1.215 (2)
C1—C2	1.398 (3)	C1A—C2A	1.387 (3)
C1—C10	1.428 (3)	C1A—C10A	1.434 (3)
C1—C11	1.457 (3)	C1A—C11A	1.457 (3)
C2—C3	1.403 (3)	C2A—C3A	1.408 (3)
C2—H2	0.9500	C2A—H2A	0.9500
C3—C4	1.429 (3)	C3A—C4A	1.431 (3)
C3—C13	1.447 (3)	C3A—C13A	1.446 (3)
C4—C5	1.391 (3)	C4A—C5A	1.394 (3)
C4—C10	1.467 (3)	C4A—C10A	1.463 (2)
C5—C6	1.387 (3)	C5A—C6A	1.383 (3)
C5—H5	0.9500	C5A—H5A	0.9500
C6—C7	1.390 (3)	C6A—C7A	1.392 (3)
C6—H6	0.9500	C6A—H6A	0.9500
C7—C8	1.386 (3)	C7A—C8A	1.390 (3)
C7—H7	0.9500	C7A—H7A	0.9500
C8—C9	1.386 (3)	C8A—C9A	1.390 (3)
C8—H8	0.9500	C8A—H8A	0.9500
C9—C10	1.389 (3)	C9A—C10A	1.389 (3)
C9—H9	0.9500	C9A—H9A	0.9500
C11—C12	1.536 (3)	C11A—C12A	1.541 (3)
C13—C14	1.544 (3)	C13A—C14A	1.546 (3)
			
C2—C1—C10	108.52 (16)	C2A—C1A—C10A	108.39 (16)
C2—C1—C11	125.49 (18)	C2A—C1A—C11A	125.81 (17)
C10—C1—C11	125.99 (17)	C10A—C1A—C11A	125.78 (17)
C1—C2—C3	109.69 (17)	C1A—C2A—C3A	110.12 (17)
C1—C2—H2	125.2	C1A—C2A—H2A	124.9
C3—C2—H2	125.2	C3A—C2A—H2A	124.9
C2—C3—C4	108.25 (16)	C2A—C3A—C4A	107.87 (16)
C2—C3—C13	125.54 (18)	C2A—C3A—C13A	125.59 (18)
C4—C3—C13	126.20 (17)	C4A—C3A—C13A	126.53 (17)
C5—C4—C3	125.52 (17)	C5A—C4A—C3A	125.60 (17)
C5—C4—C10	127.61 (17)	C5A—C4A—C10A	127.54 (17)
C3—C4—C10	106.84 (16)	C3A—C4A—C10A	106.85 (15)
C6—C5—C4	128.73 (18)	C6A—C5A—C4A	128.50 (18)
C6—C5—H5	115.6	C6A—C5A—H5A	115.7
C4—C5—H5	115.6	C4A—C5A—H5A	115.7
C5—C6—C7	128.88 (19)	C5A—C6A—C7A	129.35 (18)
C5—C6—H6	115.6	C5A—C6A—H6A	115.3
C7—C6—H6	115.6	C7A—C6A—H6A	115.3
C8—C7—C6	129.13 (19)	C8A—C7A—C6A	129.10 (19)
C8—C7—H7	115.4	C8A—C7A—H7A	115.5
C6—C7—H7	115.4	C6A—C7A—H7A	115.5
C7—C8—C9	129.54 (19)	C7A—C8A—C9A	128.92 (18)
C7—C8—H8	115.2	C7A—C8A—H8A	115.5
C9—C8—H8	115.2	C9A—C8A—H8A	115.5
C10—C9—C8	128.27 (18)	C10A—C9A—C8A	128.53 (18)
C10—C9—H9	115.9	C10A—C9A—H9A	115.7
C8—C9—H9	115.9	C8A—C9A—H9A	115.7
C9—C10—C1	125.46 (17)	C9A—C10A—C1A	125.14 (17)
C9—C10—C4	127.81 (18)	C9A—C10A—C4A	128.04 (17)
C1—C10—C4	106.65 (16)	C1A—C10A—C4A	106.74 (15)
O1—C11—C1	125.8 (2)	O1A—C11A—C1A	126.36 (19)
O1—C11—C12	117.38 (18)	O1A—C11A—C12A	117.31 (17)
C1—C11—C12	116.78 (17)	C1A—C11A—C12A	116.31 (17)
F2—C12—F1	107.72 (17)	F2A—C12A—F1A	107.42 (16)
F2—C12—F3	107.00 (17)	F2A—C12A—F3A	107.06 (16)
F1—C12—F3	107.28 (17)	F1A—C12A—F3A	107.55 (16)
F2—C12—C11	111.20 (17)	F2A—C12A—C11A	111.26 (16)
F1—C12—C11	110.51 (17)	F1A—C12A—C11A	110.94 (16)
F3—C12—C11	112.90 (17)	F3A—C12A—C11A	112.37 (16)
O2—C13—C3	125.94 (19)	O2A—C13A—C3A	126.57 (19)
O2—C13—C14	116.69 (17)	O2A—C13A—C14A	116.71 (17)
C3—C13—C14	117.37 (17)	C3A—C13A—C14A	116.72 (17)
F6—C14—F5	107.30 (16)	F6A—C14A—F4A	107.38 (17)
F6—C14—F4	107.41 (16)	F6A—C14A—F5A	107.43 (16)
F5—C14—F4	106.68 (16)	F4A—C14A—F5A	106.64 (17)
F6—C14—C13	111.59 (16)	F6A—C14A—C13A	111.71 (17)
F5—C14—C13	111.68 (16)	F4A—C14A—C13A	111.30 (16)
F4—C14—C13	111.89 (16)	F5A—C14A—C13A	112.10 (16)
			
C10—C1—C2—C3	−2.2 (2)	C10A—C1A—C2A—C3A	−1.5 (2)
C11—C1—C2—C3	178.56 (18)	C11A—C1A—C2A—C3A	179.96 (17)
C1—C2—C3—C4	1.6 (2)	C1A—C2A—C3A—C4A	1.8 (2)
C1—C2—C3—C13	−178.33 (18)	C1A—C2A—C3A—C13A	−177.22 (17)
C2—C3—C4—C5	177.72 (17)	C2A—C3A—C4A—C5A	177.00 (18)
C13—C3—C4—C5	−2.3 (3)	C13A—C3A—C4A—C5A	−4.0 (3)
C2—C3—C4—C10	−0.4 (2)	C2A—C3A—C4A—C10A	−1.4 (2)
C13—C3—C4—C10	179.51 (18)	C13A—C3A—C4A—C10A	177.65 (18)
C3—C4—C5—C6	−177.32 (19)	C3A—C4A—C5A—C6A	−177.74 (18)
C10—C4—C5—C6	0.4 (3)	C10A—C4A—C5A—C6A	0.3 (3)
C4—C5—C6—C7	1.3 (3)	C4A—C5A—C6A—C7A	−0.3 (3)
C5—C6—C7—C8	−0.6 (4)	C5A—C6A—C7A—C8A	1.2 (3)
C6—C7—C8—C9	−1.1 (4)	C6A—C7A—C8A—C9A	−1.5 (3)
C7—C8—C9—C10	0.9 (4)	C7A—C8A—C9A—C10A	0.4 (3)
C8—C9—C10—C1	177.52 (19)	C8A—C9A—C10A—C1A	177.21 (18)
C8—C9—C10—C4	1.1 (3)	C8A—C9A—C10A—C4A	0.9 (3)
C2—C1—C10—C9	−175.17 (18)	C2A—C1A—C10A—C9A	−176.37 (17)
C11—C1—C10—C9	4.1 (3)	C11A—C1A—C10A—C9A	2.2 (3)
C2—C1—C10—C4	1.8 (2)	C2A—C1A—C10A—C4A	0.6 (2)
C11—C1—C10—C4	−178.89 (18)	C11A—C1A—C10A—C4A	179.14 (17)
C5—C4—C10—C9	−2.0 (3)	C5A—C4A—C10A—C9A	−1.0 (3)
C3—C4—C10—C9	176.06 (18)	C3A—C4A—C10A—C9A	177.34 (18)
C5—C4—C10—C1	−178.95 (18)	C5A—C4A—C10A—C1A	−177.85 (18)
C3—C4—C10—C1	−0.9 (2)	C3A—C4A—C10A—C1A	0.47 (19)
C2—C1—C11—O1	−171.8 (2)	C2A—C1A—C11A—O1A	−168.50 (19)
C10—C1—C11—O1	9.0 (3)	C10A—C1A—C11A—O1A	13.2 (3)
C2—C1—C11—C12	9.7 (3)	C2A—C1A—C11A—C12A	13.2 (3)
C10—C1—C11—C12	−169.47 (18)	C10A—C1A—C11A—C12A	−165.03 (17)
O1—C11—C12—F2	3.9 (3)	O1A—C11A—C12A—F2A	−1.3 (3)
C1—C11—C12—F2	−177.52 (17)	C1A—C11A—C12A—F2A	177.14 (16)
O1—C11—C12—F1	−115.7 (2)	O1A—C11A—C12A—F1A	−120.81 (19)
C1—C11—C12—F1	62.9 (2)	C1A—C11A—C12A—F1A	57.6 (2)
O1—C11—C12—F3	124.1 (2)	O1A—C11A—C12A—F3A	118.75 (19)
C1—C11—C12—F3	−57.2 (2)	C1A—C11A—C12A—F3A	−62.8 (2)
C2—C3—C13—O2	168.8 (2)	C2A—C3A—C13A—O2A	175.72 (19)
C4—C3—C13—O2	−11.1 (3)	C4A—C3A—C13A—O2A	−3.1 (3)
C2—C3—C13—C14	−10.9 (3)	C2A—C3A—C13A—C14A	−4.6 (3)
C4—C3—C13—C14	169.21 (17)	C4A—C3A—C13A—C14A	176.51 (17)
O2—C13—C14—F6	6.2 (3)	O2A—C13A—C14A—F6A	0.0 (3)
C3—C13—C14—F6	−174.04 (16)	C3A—C13A—C14A—F6A	−179.65 (17)
O2—C13—C14—F5	−113.9 (2)	O2A—C13A—C14A—F4A	120.0 (2)
C3—C13—C14—F5	65.9 (2)	C3A—C13A—C14A—F4A	−59.6 (2)
O2—C13—C14—F4	126.63 (19)	O2A—C13A—C14A—F5A	−120.6 (2)
C3—C13—C14—F4	−53.7 (2)	C3A—C13A—C14A—F5A	59.7 (2)

### Hydrogen-bond geometry (Å, °)

**Table:**

*D*—H···*A*	*D*—H	H···*A*	*D*···*A*	*D*—H···*A*
C2—H2···F3	0.95	2.40	2.916 (2)	114
C2A—H2A···F3A	0.95	2.45	2.972 (2)	115
C2A—H2A···F5A	0.95	2.50	2.968 (2)	111
C5—H5···O2	0.95	2.31	2.988 (2)	127
C5A—H5A···O2A	0.95	2.33	3.001 (2)	127
C6—H6···O2A^i^	0.95	2.52	3.236 (2)	133
C6A—H6A···O2^i^	0.95	2.47	3.160 (2)	130
C8—H8···F2A^ii^	0.95	2.45	3.358 (2)	160
C9—H9···O1	0.95	2.31	2.983 (2)	127
C9—H9···O1A^ii^	0.95	2.58	3.454 (2)	153
C9A—H9A···O1^ii^	0.95	2.50	3.352 (2)	149
C9A—H9A···O1A	0.95	2.31	2.993 (2)	128

Symmetry codes: (i) −*x*+1, −*y*+2, −*z*; (ii) −*x*+1, −*y*+2, −*z*+1.
